# Antidiabetic Activity of* Picris japonica* Thunb Aqueous Extract in Diabetic KK-A^y^ Mice

**DOI:** 10.1155/2018/1298030

**Published:** 2018-11-12

**Authors:** Yanmei Jia, Lirong Chen

**Affiliations:** Laboratory Medicine Department, Fenyang College of Shanxi Medical University, Fenyang, 032200, China

## Abstract

**Objective:**

To evaluate the hypoglycemic effect of Picris japonica Thunb (Asteraceae) on KK-Ay mice.

**Methods:**

The hypoglycemic effect of* Picris japonica* aqueous extract (PJE) in a spontaneous type 2 diabetic model (KK-A^y^ mice) was studied in the present research. PJE was administrated at doses of 700 mg/kg and 350 mg/kg (calculated as crude herb) for 14 days and blood glucose, oral glucose tolerance test, plasma insulin level, and blood lipid were evaluated. Meanwhile, Rosiglitazone was used for the positive control.

**Results:**

It was found the PJE treatment significantly reduced blood glucose level and improved oral glucose tolerance ability (*p* < 0.01 or* p* < 0.05) in a dose-dependent manner compared to the control diabetic mice. The blood insulin levels were significantly reduced in PJE-treated mice (700 mg/kg) and Rosiglitazone compared with the diabetic control (*p* < 0.01). Compared with the control diabetic group, the serum total cholesterol, triglyceride, and low density lipoprotein cholesterol were reduced by PJE (700 mg/kg) and Rosiglitazone (*p* < 0.05), and the serum high density lipoprotein cholesterol was significantly increased only by Rosiglitazone (*p* < 0.01).

**Conclusions:**

The findings demonstrate that* Picris japonica* has remarkable antidiabetic effect in diabetic KK-A^y^ mice, which suggests that* Picris japonica* may be beneficial to the treatment of type 2 diabetes mellitus.

## 1. Introduction

Diabetes mellitus is a chronic metabolic disorder, which can be classified into two types: type 1 diabetes (insulin-dependent diabetes mellitus) and type 2 diabetes (noninsulin dependent diabetes mellitus) and the latter account for > 90% of all diabetic cases. It is estimated that more than 170 million people worldwide suffer from diabetes and the number is predicted to double to > 300 million by the year 2030 [1]. In type 2 diabetes mellitus, insulin resistance and relative deficiency of insulin secretion are two characteristic features and current clinical therapies mainly focus on increasing plasma insulin level by direct insulin administration and oral agents that promote insulin secretion, improving insulin sensitivity by drugs such as thiazolidinediones (TZDs) and delaying the digestion and absorption of carbohydrate from the gastrointestinal tract by inhibition of *α*-glucosidase [2]. Rosiglitazone (BRL49653), one of the thiazolidinedione antihyperglycemic drugs, is identified as synthetic ligand for peroxisome proliferator-activated receptor *γ* (PPAR*γ*) which is a member of the nuclear hormone receptor superfamily [3]. Rosiglitazone is used clinically to improve insulin sensitivity and lower blood glucose in diabetic patients [4, 5]. In the present study, rosiglitazone was used for the positive control.

Recently, the search for appropriate antidiabetic agents from traditional herbal medicines has been an important strategy [6–9].* Picris japonica* Thunb recorded as herbal medicine is traditionally used for the treatment of respiratory system diseases in China [10]. In some regions, it is also used for diet and the treatment of diabetic mellitus. Other data related to* Picris japonica* are very few. It was reported that* Picris japonica* aqueous extract exhibited remarkable hypoglycemic effect in alloxan-induced diabetic rat model [11]. However, KK-A^y^ mice model is one of the useful type 2 diabetic models due to its various characteristics such as hyperglycemia, glucose intolerance, insulin resistance, and increased gluconeogenesis and is widely used for evaluation of antidiabetic agents [12, 13]. So, in the present study, KK-A^y^ diabetic mice model is used to evaluate the antidiabetic effects of* Picris japonica* aqueous extract through measurement of blood glucose, glucose tolerance ability, and plasma insulin level as well as blood lipid to further provide a basis for the application of* Picris japonica* in the treatment of type 2 diabetes mellitus.

## 2. Methods

### 2.1. Preparation of Picris Japonica Extract

The whole herbal plants of* Picris japonica* were collected in Fangshan county, Shanxi Province and identified by Jia Yanmei (Fenyang College of Shanxi Medical University). A voucher specimen (2016-07) was deposited in Fenyang College of Shanxi Medical University, China.

The pieces of herbal were mixed with water (1:5, w/v) and extracted at 60°C for 2 h. The extract was filtered and freeze-dried to obtain the freeze-dried powder. Then the powder was suspended in saline to prepare 0.1 g/ml solution and stored at 4°C for use.

### 2.2. Phytochemical Detection for PJE

The phytochemical evaluation of the PJE was performed by specific procedures for the detection of total phenolic, total flavonoids, and total saponins as described in previous studies [14–16]. Gallic acid, rutin, and ursolic acid were used for analytic standards to construct the calibration curves. All experiments were carried out in triplicate and the results were presented as standard equivalents.

### 2.3. Animals

Animal experiments were carried out under principles in good laboratory animal care and approved by ethical committee for Laboratory Animals Care and Use of Fenyang College of Shanxi Medical University. KK-A^y^ mice (7 weeks old) were obtained from Institute of Laboratory Animals, Chinese Academy of Medical Sciences, Beijing, China (SCXK 2005-0013). Before and during the experiments, the mice were housed in cages and maintained in 12 h light/dark cycle at a ambient temperature (22 ± 2°C) and humidity (50 ± 10%). All mice were given a high-fat diet (GB14924.3-2001).

### 2.4. Experimental Design

After four weeks of high-fat diet feeding, the fasted blood glucose levels of the mice were tested, and the mice who were fasted blood glucose levels ≥ 11.2 mmol/L were determined as type 2 diabetes mellitus. Then, the mice were divided randomly into four groups (n = 10 for each group). Group 1, diabetic control mice fed with saline; Group 2, diabetic mice fed with 700 mg/kg PJE; Group 3, diabetic mice fed with 350 mg/kg PJE; Group 4, diabetic mice fed with 4 mg/kg Rosiglitazone (GlaxoSmithKline, Tianjing, China). Body weight was measured at 7th and 14th day. After continuous feeding for 14 days, blood samples were collected from tail vein in heparin-containing sample tubes. Then the samples were centrifuged at 4°C (6000 g, 10 min) to collect plasma which was stored at –20°C for use.

### 2.5. Blood Glucose Level

Blood glucose level was assayed by a commercial glucose kit based on the glucose oxidase method (BioSino Bio-technology and Science Inc., Beijing, China).

### 2.6. Oral Glucose Tolerance Test (OGTT)

KK-A^y^ mice were treated as described above in* Experimental design* for 12 days and then were administered orally with glucose solution (2.5 g/kg) and blood samples were collected from tail vein at 0, 30, 60, and 120 min and blood glucose levels of all blood samples were measured by a commercial glucose kit.

### 2.7. Plasma Insulin Level

After administration for 14 days, blood samples were prepared and plasma insulin level was determined by enzyme linked immunosorbent assay kit (Beijing FuRui Biological Enginering Company, Beijing, China).

### 2.8. Blood Lipid Level

After administration for 14 days, blood samples were prepared and plasma TC, TG, HDL, and LDL levels were determined by commercial assay kits (BioSino Bio-technology and Science Inc., Beijing, China).

### 2.9. Statistical Analysis

The results were expressed as mean ± SD and statistical significance was determined by one-way ANOVA followed by Dunnet t-test.* p* < 0.05 was regarded as significant difference between groups.

## 3. Results

### 3.1. Phytochemical Composition of PJE

The main composition of PJE and calibration curves parameters were presented in Table 1.

### 3.2. Effect of PJE on Body Weight in KK-A^y^ Mice

As shown in Table 2, there was no obvious increase in mice body weight during the experiment and all mice were at a normal range in body weight, which demonstrated that no obesity occurred in all mice. Meanwhile, the study found that PJE had no effect on the body weight of KK-A^y^ mice during the 14-day period of treatment compared with diabetic control mice.

### 3.3. Effect of PJE on Blood Glucose Level in KK-A^y^ Mice

**Figure 1:**
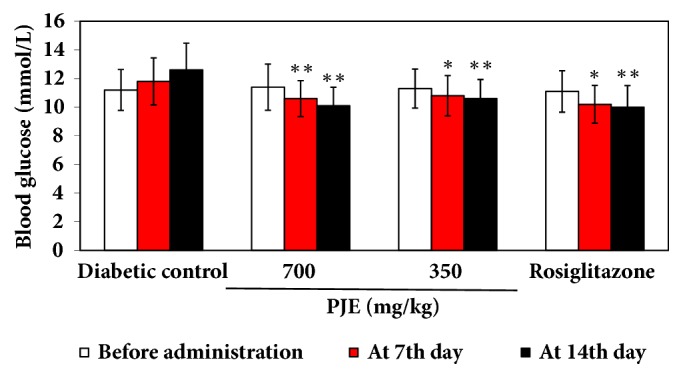
Effect of PJE on blood glucose in KK-A^y^ mice. Results were presented as mean ± SD (n =10). *∗∗p* < 0.01, compared with diabetic control; *∗p* < 0.05, compared with diabetic control.

As shown in Figure 1, the blood glucose levels in mice of different groups were maintained at around 11 mmol/L before administration. The blood glucose level in diabetic control mice increased from 11.1 to 11.7 and 12.6 mmol/L at 7th and 14th day, respectively. Oral administration of PJE for 7 and 14 days caused a significant decrease in blood glucose level in a dose-dependent manner compared with the control diabetic group (*p* < 0.05 or* p* < 0.01). Administration of Rosiglitazone also significantly decreased blood glucose level of mice compared with the control diabetic group (*p* < 0.01). There was no significant difference between the PJE groups and the Rosiglitazone group.

### 3.4. Effect of PJE on Oral Glucose Tolerance Test in KK-A^y^ Mice

**Figure 2:**
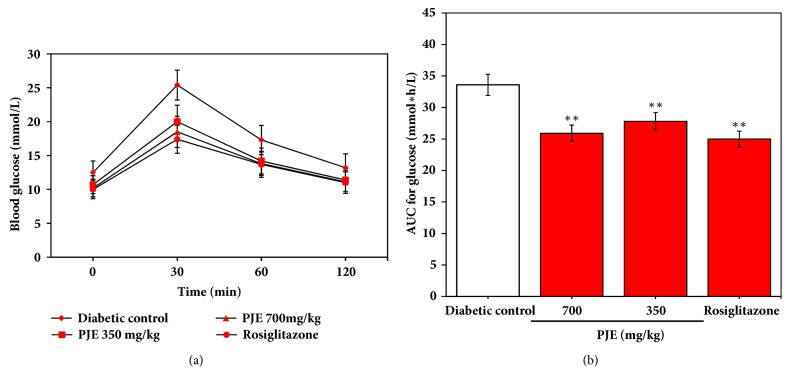
Effect of PJE on glucose tolerance in KK-A^y^ mice. (a) Establishment of oral glucose intolerance test after 12 days' treatment. Results were presented as mean ± SD (n =10). (b) Area under the glucose curve obtained at the end of the treatment. *∗∗p* < 0.01, compared with diabetic control; *∗p* < 0.05, compared with diabetic control.

As shown in Figure 2, oral administration of PJE for 12 days appeared to obviously improve the glucose tolerance of KK-A^y^ mice. The control diabetic mice showed a sharp increase from 12.5 ± 1.89 mmol/L to 24.4 ± 2.47 mmol/L in blood glucose level at 30 min after oral administration of 2.5 g/kg glucose and maintained high level of blood glucose for over an additional 30 min. All PJE-treated and Rosiglitazone-treated mice also showed sharp increases in blood glucose level at 30 and 60 min after oral administration of 2.5 g/kg glucose. However, compared with the control diabetic group, PJE- and Rosiglitazone-treated mice showed significant lower level in blood glucose (*p* < 0.01).

### 3.5. Effect of PJE on Blood Insulin Level in KK-A^y^ Mice

**Figure 3:**
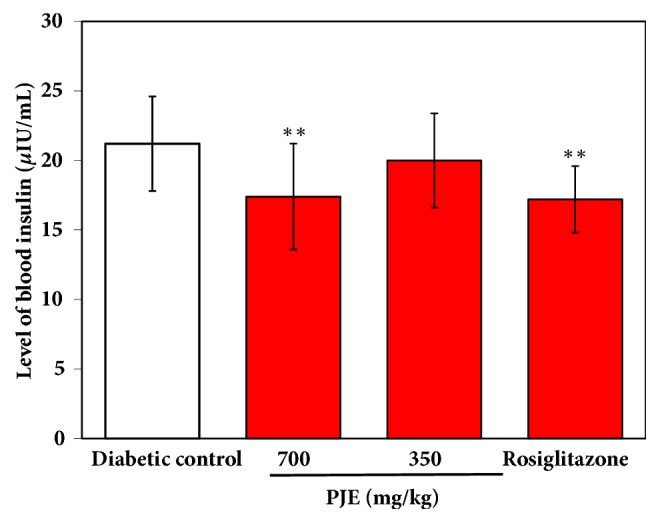
Effect of PJE on blood insulin in KK-A^y^ mice. Results were presented as mean ± SD (n = 10). *∗∗p* < 0.01, compared with diabetic control; *∗p *< 0.05, compared with diabetic control.

As shown in Figure 3, the blood insulin levels were significantly reduced in PJE-treated mice (700 mg/kg) and Rosiglitazone-treated mice compared with the diabetic control (*p* < 0.01), which illustrated that PJE can relieve the insulin tolerance in the KK-Ay mice.

### 3.6. Effect of PJE on Blood Lipid Level in KK-A^y^ Mice

As shown in Table 3, oral administration of PJE (700 mg/kg) and Rosiglitazone for 14 days showed different effects on total cholesterol, triglyceride, high density lipoprotein cholesterol, and low density lipoprotein cholesterol in KK-Ay mice fed with a high-fat diet. Compared with the control diabetic group, the serum total cholesterol, triglyceride, and low density lipoprotein cholesterol were reduced by PJE (700 mg/kg) and Rosiglitazone (*p* < 0.05), and the serum high density lipoprotein cholesterol was significantly increased only by Rosiglitazone (*p* < 0.01). PJE (350 mg/kg) exhibited no effect on blood lipid.

## 4. Discussion

The purpose of the present study was to evaluate the hypoglycemic activity of* Picris japonica* aqueous extract in KK-A^y^ diabetic mice. Hyperglycemia, insulin resistance, and dysfunction of insulin secretion are major characteristic features of type 2 diabetes mellitus. In a previous report, the alloxan-induced diabetic mice model was used to evaluate the antidiabetic effect of* Picris japonica* aqueous extract. This chemically induced diabetic model was based on the unique capability of alloxan to selectively destroy the pancreatic beta cells, which was pathologically similar to type 1 diabetes mellitus [17]. The model of KK-A^y^ mice was a spontaneously diabetic model, which was established by transferring the yellow obese gene (A^y^) into KK mice. This diabetic model was a relevant model of human type 2 diabetes mellitus due to its related changes in systemic organs and used worldwide for the assessment of pharmacological effects of new antidiabetes agents [18]. Therefore, KK-A^y^ mice were used for assessment of the antidibetic activity of* Picris japonica* in the present study to further provide evidence for the application of* Picris japonica* Thunb in type 2 diabetes mellitus.

Hyperglycemia, a principal symptom in type 2 diabetic mellitus, is widely perceived as contributing to the diabetic retinopathy and other diabetic complications. Hyperglycemia activates polyol pathway which is a two-step metabolic pathway where glucose is reduced to sorbitol first and then sorbitol is converted into fructose. Under hyperglycemic conditions, sorbitol accumulates in tissues like retina, kidney, peripheral nerves, and blood vessels where it causes osmotic damage to the tissues [19–21]. Meanwhile, hyperglycemia leads to accumulation of advanced glycation end products (AGEs) which are nonenzymatically glycated and oxidized proteins and lipids. AGEs activate protein kinase C (PKC) and poly (ADP-ribose) polymerase both of which initiative inflammation in tissues [22]. Stratton et al. also proposed that hyperglycaemia was strongly associated with the risk of diabetic complications in patients with type 2 diabetes based on a clinical study of 4585 patients [23].

Type 2 diabetes is noninsulin dependent and occurs as a result of insulin resistance and deficiency in insulin secretion. The action of insulin is to promote carbohydrate uptake and conversion to lipids. Insulin resistance refers to the condition where the insulin is not very effective in the conversion from glucose to lipid, which gives rise to high blood glucose levels. In the present study,* Picris japonica* aqueous extract significantly reduced blood glucose level, increased blood insulin level, and regulated blood lipid level compared with the control diabetic group, which indicated that* Picris japonica *maybe a potential agent for the intervention of diabetic complications.

## 5. Conclusion

The findings demonstrate that* Picris japonica* has a remarkable hypoglycemic effect in diabetic KK-A^y^ mice suggesting that* Picris japonica* may be beneficial in the treatment of type 2 diabetes mellitus.

## Figures and Tables

**Table 1:** Results and calibration curves parameters of phytochemical evaluation of PJE.

Assay	Analytic standard	Curve parameters (Equation/R^2^)	Content (mg equivalents / 100 g extract)
Total phenolics	Gallic acid	y = 101.3x + 0.00079 / R^2^ = 0.998	21.35 ± 1.23
Total flavonoids	Rutin	y = 12.008x - 0.0115 / R^2^ = 0.997	3725.21 ± 0.04
Total saponins	Ursolic acid	y = 4.132x - 0.0015 / R^2^ = 0.998	492.47 ± 0.28

**Table 2:** Effect of PJE on body weight (g) in KK-A^y^ mice. Results were presented as mean ± SD (n = 10). *∗∗p* < 0.01, compared with diabetic control; *∗p* < 0.05, compared with diabetic control.

	Day 1	Day 7	Day 14
Diabetic control	29.4 ± 2.31	31.5 ± 2.11	32.2 ± 2.61
PJE (700 mg/kg)	29.8 ± 2.40	30.2 ± 2.44	31.4 ± 2.23
PJE (350 mg/kg)	29.1 ± 2.22	31.4 ± 2.87	32.8 ± 2.47
Rosiglitazone	30.4 ± 2.51	32.1 ± 2.79	31.0 ± 2.39

**Table 3:** Effect of PJE on blood lipid in KK-A^y^ mice. Results were presented as mean ± S.D (n = 10). *∗∗p* < 0.01, compared with diabetic control; *∗p* < 0.05, compared with diabetic control.

	Total Cholesterol (mmol/L)	Triglyceride (mmol/L)	High density lipoprotein cholesterol (mmol/L)	Low density lipoprotein cholesterol (mmol/L)
Diabetic control	3.86 ± 0.28	3.32 ± 0.27	0.75 ± 0.06	1.84 ± 0.21
PJE (700 mg/kg)	2.92 ± 0.31*∗*	2.78 ± 0.21*∗*	0.81 ± 0.08	1.59 ± 0.24*∗*
PJE (350 mg/kg)	3.41 ± 0.25	3.05 ± 0.23	0.78 ± 0.08	1.76 ± 0.22
Rosiglitazone	2.87 ± 0.27*∗*	1.92 ± 0.21*∗∗*	0.91 ± 0.07*∗∗*	1.64 ± 0.19*∗*

## Data Availability

The data used to support the findings of this study are available from the corresponding author upon request.
